# *Toxoplasma* encephalitis in an HIV-positive patient – a case report

**DOI:** 10.1186/1471-2334-13-S1-P9

**Published:** 2013-12-16

**Authors:** Adela Burac, Florin Terteliu, Corina Stănescu

**Affiliations:** 1 District Hospital of Suceava, Romania

## Background

Toxoplasmosis still represents the most frequent complication affecting the CNS of patients with AIDS.

## Case report

We present a case of *Toxoplasma* encephalitis in a 23 year-old patient with HIV infection, with fatal outcome. For diagnosis we used neuroimaging methods (CT and MRI) that demonstrated multiple masses surrounded by edema and central-necrotic arias) and serum *Toxoplasma* IgG and IgM – high levels. Under co-trimoxazol treatment there was an improvement of the lesions on neuroimaging, in contrast with the worsening of the symptoms, convulsions and fatal outcome.

